# Relation between myocardial edema and myocardial mass during the acute and convalescent phase of myocarditis – a CMR study

**DOI:** 10.1186/1532-429X-10-19

**Published:** 2008-04-30

**Authors:** Anja Zagrosek, Ralf Wassmuth, Hassan Abdel-Aty, André Rudolph, Rainer Dietz, Jeanette Schulz-Menger

**Affiliations:** 1 Franz-Volhard-Klinik, Charité-Campus Buch, Humboldt-University Berlin, Helios-Klinikum, Germany

## Abstract

**Background:**

Myocardial edema is a substantial feature of the inflammatory response in human myocarditis. The relation between myocardial edema and myocardial mass in the course of healing myocarditis has not been systematically investigated. We hypothesised that the resolution of myocardial edema as visualised by T2-weighted cardiovascular magnetic resonance (CMR) is associated with a decrease of myocardial mass in steady state free precession (SSFP)-cine imaging.

**Methods:**

21 patients with acute myocarditis underwent CMR shortly after onset of symptoms and 1 year later. For visualization of edema, a T2-weighted breath-hold black-blood triple-inversion fast spin echo technique was applied and the ratio of signal intensity of myocardium/skeletal muscle was assessed. Left ventricular (LV) mass, volumes and function were quantified from biplane cine steady state free precession images.

11 healthy volunteers served as a control group for interstudy reproducibility of LV mass.

**Results:**

In patients with myocarditis, a significant decrease in LV mass was observed during follow-up compared to the acute phase (156.7 ± 30.6 g vs. 140.3 ± 28.3 g, p < 0.0001). The reduction of LV mass paralleled the normalization of initially increased myocardial signal intensity on T2-weighted images (2.4 ± 0.4 vs. 1.68 ± 0.3, p < 0.0001).

In controls, the interstudy difference of LV mass was lower than in patients (5.1 ± 2.9 g vs. 16.3 ± 14.2 g, p = 0.02) resulting in a lower coefficient of variability (2.1 vs 8.9%, p = 0.04).

**Conclusion:**

Reversible abnormalities in T2-weighted CMR are paralleled by a transient increase in left ventricular mass during the course of myocarditis. Myocardial edema may be a common pathway explaining these findings.

## Introduction

An array of reports demonstrated the linear correlation between myocardial water content and T2-weighted cardiovascular magnetic resonance (CMR) relaxation [1-7], justifying its use as an in-vivo marker of myocardial edema. Myocardial edema is a substantial feature of the inflammatory response in acute myocarditis[8,9]. Edema is associated with diastolic dysfunction[10], conduction disturbances[11], microvascular compression[12] and tissue swelling[9]. The relation between myocardial edema and myocardial mass over the course of myocarditis has only rarely been systematically investigated in a clinical setting [13,14]. This reflected the lack of a non-invasive imaging modality allowing the in vivo, accurate and simultaneous measurement of both parameters.

Indeed, histopathological quantification of edema is challenging. Over the last years, CMR has also become an important tool for the non-invasive assessment of myocarditis [15-19]. On the other hand, steady state free precession (SSFP) CMR is the current gold standard to measure myocardial mass[20] and detects even minor changes during the course of diseases [21-25] or in response to therapy. Sample size for studies of cardiac remodeling can be reduced with the use of CMR[26]. Based on these considerations we attempted to explore the relation between myocardial mass and T2-weighted CMR-imaging in a group of myocarditis patients during the acute and the convalescent phases of the disease.

We hypothesised that the resolution of myocardial edema in the course of healing myocarditis as visualised by T2-weighted CMR is associated with a measurable decrease in myocardial mass in SSFP-cine CMR.

## Methods

### Patients and controls

A group of 21 patients with clinically suspected acute myocarditis presenting to the emergency room was included after CMR confirmed the diagnosis of acute myocarditis. 16 of these patients have already been described in a previous publication and were included retrospectively[17]. 5 consecutive patients were included prospectively. For inclusion, patients had to fulfill each of the following clinical criteria:

- new onset of chest pain or shortness of breath

- history of acute infection within the last few weeks

- arrhythmias and/or pathological ECG findings

- elevation of cardiac serum markers (creatine kinase or troponin T or I)

- exclusion of coronary artery disease by conventional coronary angiography, except for patients <26 years with low pre-test probability for coronary artery disease

Exclusion criteria were clinical evidence of chronic myocarditis (history, ECG changes suggestive of former myocarditis, previous positive CMR study, etc.), previous myocardial infarction and contraindications for CMR (automatic implantable defibrillators, pacemakers, intracranial aneurysm clip, etc.).

A control group of 11 healthy volunteers was scanned twice in the course of 1 week to determine the interstudy reproducibility of LV parameters in cine-SSFP imaging.

All participants gave informed written consent including retrospective analysis of data and the study was approved by the local ethics committee.

The acute CMR scan took place as soon as possible after the patients' admission to our institution (2.6 ± 3.1 days, mean ± SD). The follow-up scan was scheduled for 1 year after, with some patients being referred for follow-up by their physicians even earlier. At follow-up, patients were asked to fill out questionnaires regarding heart failure symptoms, cardiac arrhythmias and medical treatment.

### CMR

All participants were examined using a 1.5 Tesla CMR scanner (Signa CV/*i*, GE Healthcare Technologies, Milwaukee, WI, USA). Localization was performed using breath-hold real time and steady-state free precession images of true anatomical axes of the heart. Breath-hold cine SSFP gradient echo images were acquired in a midventricular short axis, two- and four chamber view using a four-element phased array cardiac coil. Field of view was typically 380 × 380 mm, matrix size was 256 × 192 resulting in an in-plane resolution of 2 × 1.5 mm/pixel. Slice thickness was 8 mm.

For assessment of edema a T2-weighted breath-hold black-blood triple-inversion fast spin echo technique was used (TR = 2RR, TE 64 ms, IT 140 ms, slice thickness 15 mm, matrix 256 × 256, field of view 34–38 cm) and images were obtained in 3 short axis slices using the body coil.

For assessment of the global relative enhancement (gRE) a T1-weighted, ECG-triggered, free breathing spin echo sequence in four identical axial slices both before and after intravascular injection of 0.1 mmol gadolinium-diethylenetriaminepentaacetate (Gd-DTPA, Magnevist, Schering, Germany) was applied (TR 480 to 725 ms, TE 30 ms, slice thickness 6 mm, matrix size 256 × 256, body coil). Measurements after Gd-DTPA were started immediately after injection (using an automated injector [Medrad, Indianola, Pennsylvania]) and lasted 3 to 4 min. (without any change in parameters in between).

After the acquisition of spin echo images, for acquisition of late gadolinium enhancement (LGE) images, an additional dose (0.1 mmol) of Gd-DTPA was injected, and a breathhold contrast-enhanced inversion-recovery gradient-echo sequence (TR 5.5 ms, TE 1.4 ms, TI 225 to 275 ms as individually optimized to null myocardial signal, slice thickness/gap 15/5 mm, matrix 256 × 192) was applied after a delay of 10 min in a stack of short axis slices.

The diagnosis of acute myocarditis was obtained by combining the clinical criteria described above with the CMR findings. Acute myocarditis according to CMR was diagnosed when "any-two" of the three sequences described above (T2-weighted sequences, gRE and LGE) was positive (as previously described by Abdel-Aty [17])

#### Coronary angiography

Conventional X-ray coronary angiography was performed before the CMR examination by experienced cardiologists on a standard angiography suite (Hicor, Siemens, Erlangen, Germany).

### Image analysis

For quantification of myocardial edema in T2-weighted CMR, the ratio of mean signal intensity of the myocardium compared to that of the skeletal muscle was used (T2 ratio, as described elsewhere [18]). gRE was calculated as previously described by Friedrich et al[18]. LGE was analysed for the presence, number and transmurality of areas of focal high signal intensity.

Left ventricular (LV) mass, volume and function was measured in a biplanar area-length-method including two- and four-chamber views (MASS 6, Medis, Leiden, the Netherlands). LV endocardial and epicardial borders were traced manually in both, papillary muscles were not traced and thus excluded from LV mass measurement. LV mass was calculated by multiplication of LV volumes by the factor of 1.05 (specific gravity of the myocardium). LV ejection fraction was calculated as EF = ([LVEDV-LVESV]/LVEDV).

Image analysis was done by one investigator blinded to the patients' clinical data.

### Statistical analysis

The statistical tests were performed using a commercially available statistical program (SPSS 11 for Macintosh, SPSS GmbH Software, Munich, Germany). Continuous variables were compared using a paired student' t-test since all of the variables were normally distributed as assessed by a Kolmogorov-Smirnov test. Data were correlated using the Spearman or Pearson correlation coefficients depending on their distribution pattern. The coefficient of variability (percentage) was calculated as the SD of the differences in relation to the mean of the parameter under investigation.

Data are presented as mean ± standard deviation (SD). A p-value of <0.05 was considered statistically significant.

## Results

The group of patients with acute myocarditis consisted of 16 men and 5 women with a mean age of 36.4 ± 16.1 years (mean ± SD) presenting to the emergency room. Initially, 76% of the patients (16/21) received a beta blocker and 95% an ACE-Inhibitor (20/21). Medication was continued in a substantial number of patients (beta blocker 43% [9/21], ACE-inhibitor 52% [11/21]) until after the follow-up CMR scan. At follow-up, all patients were free of symptoms and had no clinical evidence of ongoing inflammation. Blood pressure values during both CMR exams were within normal ranges (see table 1 for patients' characteristics). In 16 patients (76%) the presence of significant coronary artery disease (> 50% stenosis) was excluded by conventional X-ray coronary angiography. 3 of these patients (14%) were younger than 26 years and underwent this procedure because of their history of smoking. 5 patients (24%) did not undergo this procedure due to their age <26 years and a low pre-test probability of coronary artery disease (absence of cardiovascular risk factors, age ranging from 16 to 26 years with a mean age of 20.6 ± 3.6 years).

**Table 1:** Patients' characteristics

**Nr**	**Gender**	**Age**	**CAD excluded invasively**	**ECG**	**Ck (U/l)**	**Troponin (ug/l)**	**BP (mmHg)**	**BP-FU (mmHg)**	**Initial medication**	**Medication at FU**
1	Male	20	no	ST-Elevation II, III, aVF, V3-5 T-Inversion I, aVL	443	Troponin I 39.0	125/75	110/60	Metoprolol, Ramipril	Metoprolol, Ramipril
2	Male	36	yes	ST-Elevation V5–V9 T-Inversion III, aVF	407	Troponin I 8.5	138/74	146/92	Metoprolol, Ramipril	None
3	Male	50	yes	ST-Elevation I, II, aVF, V5-6	609	Troponin I 70.0	105/70	n/a	Metoprolol, Ramipril	None
4	Female	50	yes	ST-Elevation V1, V2 T-Inversion I, II, aVL, aVF, V2–V6		Troponin I 1.3	120/70	117/49	Ramipril	Ramipril
5	Male	38	yes	ST-Elevation II, III, aVF, V5-6	213	Troponin I 15.7	130/75	110/80	Ramipril	None
6	Female	66	yes	T-Inversion I, II, aVL, V2-6	547	Troponin T 1.8	118/73	122/70	Metoprolol, Ramipril	Metoprolol, Ramipril
7	Male	51	yes	ST-Elevation V2-5 T-Inversion III	349	Troponin T 1.6	n/a	160/96	Metoprolol, Ramipril	Metoprolol
8	Female	24	yes	AV-Block III	125	Troponin T 0.2	120/60	112/51	Metoprolol, Candesartan	None
9	Male	35	yes	ST-Elevation I, II, aVF, V1-3, V5-6	166	Troponin T 0.22	120/75	n/a	Ramipril	Ramipril
10	Male	26	no	ST-Elevation I, II, aVF, V4–V6	85	Troponin I 2.0	125/80	120/80	Metoprolol, Ramipril	Metoprolol, Ramipril
11	Male	37	yes	ST-Elevation I, II, aVL, V4-6	1100	Troponin I 103.0	120/75	115/70	Metoprolol, Ramipril	Metoprolol, Ramipril
12	Female	63	yes	ST-Elevation I, II, aVL, V5-6 T-Inversion III	429	Troponin I 70.1	130/75	100/60	Metoprolol, Ramipril	Ramipril
13	Male	18	yes	ST-Elevation II, III, aVF, V5-6 T-Inversion II, III, aVF, V5-6	168	Troponin I 41.0	120/70	122/56	Metoprolol, Ramipril	None
14	Male	25	yes	ST-Elevation II, III, aVF	1767	Troponin I 198.0	115/70	130/80	Metoprolol, Ramipril	Metoprolol
15	Male	40	yes	ST-Elevation I, II, aVL, V5-6 T-Inversion III	594	Troponin T 1.0	n/a	n/a	Metoprolol, Ramipril	None
16	Male	18	no	initially Afib., later ST-Elevation II, aVF, V3-6	148	Troponin I 0.4	120/70	120/70	Metoprolol, Ramipril	Metoprolol, Ramipril
17	Male	67	yes	ST-Elevation II, III, aVF, V4-6	213	Troponin I 0.4	125/75	130/80	Metoprolol, Ramipril	None
18	Female	43	yes	T-Inversion V1–V3	205	Troponin I 0.4	120/75	130/80	Metoprolol, Ramipril	Metoprolol, Ramipril
19	Male	18	yes	ST-Elevation I, II	52	Troponin I 0.4	120/70	125/95	Metoprolol, Ramipril, Torasemid	Metoprolol, Ramipril
20	Male	16	no	ST-Elevation II, III, aVF	82	Troponin I 0.3	120/70	130/80	Ramipril	Ramipril
21	Male	23	no	ST-Elevation I, II, aVF, V2-5 T-Inversion III	570	Troponin I 0.3	120/60	124/72	None	None

Patients' clinical data at admission (Ck: creatine kinase, CAD: coronary artery disease, BP: blood pressure, FU: follow-up)

The control group consisted of 7 men and 4 female (age 31.6 ± 2.8 years, range 28–37 yrs.).

**Figure 1:**
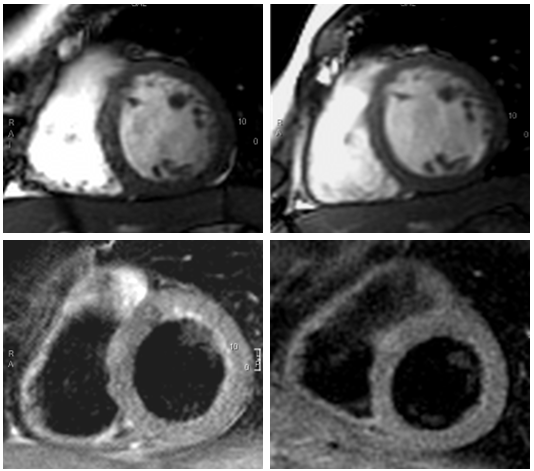
CMR images in the acute (left) and convalescent (right) phase of myocarditis. Upper left: SSFP-cine images showing increased LV mass compared to follow-up (upper right). Lower left: T2-weighted STIR images with higher signal intensity of myocardium compared to follow-up (lower right). [SSFP: steady-state free precession].

**Figure 2:**
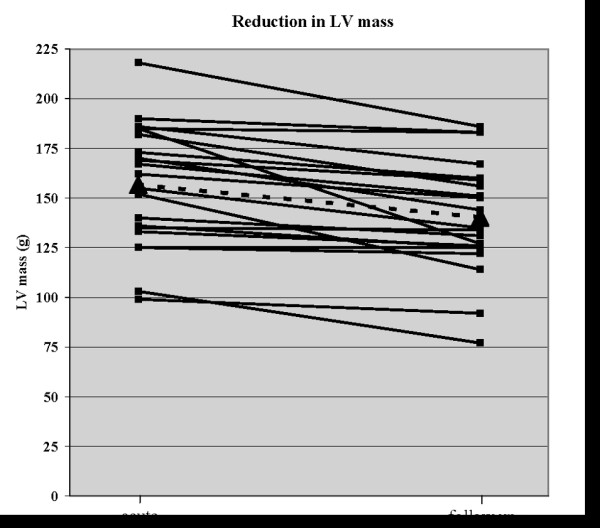
LV mass in cine-CMR in the acute and convalescent phase (follow-up) of myocarditis (dotted line: mean values of LV mass).

In patients, the LV mass in the presence of acute myocarditis was not overt hypertrophic but still within normal values at our institution. LV mass decreased significantly from 156.7 ± 30.6 g in the acute phase to 140.3 ± 28.3 g in the convalescent phase (p < 0.0001, see also figure 1 and 2). The average decrease of LV mass in the group of patients was larger than the interstudy variability in biplanar quantification of LV mass in our control group (16.3 ± 13.9 g vs. 5.1 ± 2.9 g, p = 0.02, see table 2). The left ventricular function (LV-EF) in cine imaging was within normal ranges in 18 patients (86%). However, LV-EF improved slightly but significantly in most patients at follow-up compared to the initial examination. The LV end-diastolic volume (LVEDV) did not change during the follow up period (quantitative results are given in table 3).

**Table 2:** Results of repeated cine-CMR

**Variable**	**1st examination**	**2nd examination**	**Difference between means**	**P-value 1st vs. 2nd examination**
**LV mass (g)**	140.90 ± 31.50	139.64 ± 31.00	1.30	0.50
**LV mass/height (g/cm)**	0.77 ± 0.15	0.78 ± 0.13	0.01	0.34
**LVEDV (ml)**	176.49 ± 29.13	174.36 ± 31.11	2.09	0.43
**LVEDV/height (ml/cm)**	0.97 ± 0.16	0.96 ± 0.15	0.01	0.34
**Ejection fraction (%)**	61.36 ± 3.70	61.73 ± 3.41	0.36	0.60
**T2 ratio**	1.70 ± 0.20	1.64 ± 0.21	0.06	0.65

Quantitative results of repeated cine-CMR in volunteers (mean ± SD): LV mass, LV mass/height, LVEDV, LVEDV/height, ejection fraction, T2 ratio

**Table 3:** CMR results in acute myocarditis and at follw-up

***Variable***	***Acute***	***Follow-up***	***Difference between means***	***P-value acute vs. follow-up***
**LV mass (g)**	156.66 ± 30.56	140.33 ± 28.30	-16.33	<0.0001
**LV mass/height (g/cm)**	0.90 ± 0.15	0.80 ± 0.12	-0.10	0.0001
**LVEDV (ml)**	158.10 ± 40.01	153.57 ± 37.50	-4.52	0.395
**LVEDV/height (ml/cm)**	0.89 ± 0.19	0.85 ± 0.19	-0.04	0.2859
**Ejection fraction (%)**	59.95 ± 6.39	64.14 ± 5.26	4.19	0.015
**T2 ratio**	2.41 ± 0.39	1.68 ± 0.29	-0.72	<0.0001

Quantitative results of CMR in the acute and convalescent phase (follow-up) of myocarditis (mean ± SD): LV mass, LV mass/height, LVEDV, LVEDV/height, ejection fraction, T2 ratio

The observed reduction of LV mass in patients paralleled the normalization of initially increased myocardial signal intensity on T2-weighted images. However, no quantitative correlation was found between reduction of myocardial mass (10.2 ± 8.3%) and decrease of T2 ratio (28.5 ± 14.3%, p = 0.55) in the course of myocarditis.

11 patients (52%) had positive findings on LGE images at presentation and at follow up. There were however no significant differences in the severity of LGE findings (number or transmurality). Furthermore no correlation was found between the presence of LGE and LV mass reduction (p = 0.35) or LV mass at presentation (p = 0.86).

All of the patients had elevated cardiac serum markers (creatine kinase or troponin T or I) at presentation. We found no quantitative correlation between creatine kinase (p = 0.80) or elevation of troponin (p = 0.45) and LV mass.

## Discussion

This study describes a reduction of LV mass in SSFP-cine CMR during the course of healing myocarditis coming along with a normalization of initially increased values in T2-weighted CMR.

Hiramitsu et al[13] described 25 cases of biopsy proven acute myocarditis with a transient thickening of both the interventricular septum and left ventricular wall which was associated with the presence of histologically confirmed interstitial edema. The ventricular wall thickening as well as the edema resolved in the convalescent phase of the disease. These results are also supported by several echocardiographic reports describing a transient LV hypertrophy during inflammation[14,27]. Following the conclusions of Hiramitsu et al., we do think that the observed decrease in LV mass in our study is comparable to their data and most likely reflects the regression of myocardial edema although we are not able to verify this finding with histological data.

Schroeder et al. [28] assessed 51 patients with acute myocardial infarction (AMI) and contemporary coronary reperfusion therapy for global and regional myocardial remodeling during the first year after AMI. They described an increased LV mass one week after AMI with a significant reduction in LV mass and LV wall thickness 6 months after AMI which was strongly associated with the size of infarction as assessed by peak creatine kinase-MB (CK-MB). The authors ascribe their observation of increased LV mass shortly after AMI to myocardial edema, because experimental studies have shown early interstitial edema of infarcted myocardium and increased LV mass [29] and animal studies by magnetic resonance imaging confirm these findings[1,2,4]. The article of Schroeder et al. supports the theory that myocardial edema leads to a measurable increase in LV mass in CMR.

The reduction of LV mass described in this report may in theory be a result of medical therapy rather than regression of edema. Due to the long time interval between the two CMR examinations, the observed reduction of LV mass might be an effect of medication with ACE-Inhibitors or β-blockers which were received by most patients. However, the initial LV mass was not hypertrophic but in the upper range of the normal values and none of the patients was hypertensive. Furthermore, the dosage of ramipril and metoprolol was rather low compared to the usual dosages described for regression of LV hypertrophy [30-32]. Since to our knowledge no decremental effect of therapy with ACE-Inhibitors or β-blockers on LV mass in non-hypertrophic non-hypertonic hearts is reported, we tend to believe that the reduction of LV mass in our study comes secondary to regressive edema.

In our study, the reduction of LV mass was strongly associated with a decrease of signal intensity on T2-weighted images (T2-ratio), although we were not able to find a quantitative correlation of both parameters. Higgins et al. as well as many other authors [1-5] describe a linear correlation between myocardial water content and T2-weighted cardiovascular magnetic resonance (CMR) relaxation. They found that despite the linear correlation, small changes of myocardial water content result in large T2-changes which may explain the relatively low difference of LV mass compared with larger changes in T2-ratio in the present study. The inversion recovery sequence we used is in fact not purely T2-weighted but is sensitive to tissues with long T1-relaxation times as well. This may have acted to 'amplify' the effect of free water – known to have long T1 and long T2 weighted relaxation times-resulting in a *water-weighted *image.

## Limitations

Left ventricular mass was not analyzed in contiguous short axis stacks that are considered the gold standard. Nevertheless we used 2D measurements for pairwise intraindividual comparisons both in patients and in controls. Previous studies found differences in LV mass between 2D and 3D measurements to be small[33]. In these studies, 2D quantification underestimated LV mass compared to 3D methods, thereby the true effect of tissue swelling might even be larger than reported.

We are well aware that the lack of endomyocardial biopsies is a certain limitation to our study. To ascertain the diagnosis of acute myocarditis we preferred a clinical approach. This is due to two reasons: First, the sensitivity of endomyocardial biopsy to diagnose myocarditis is limited and remains a controversial issue[34]. Second, our cohort of patients was rather young, presenting acutely with typical signs suggestive of myocarditis although none of them was severly ill in terms of heart failure. All patients revealed a preserved ejection fraction, there was no clinical need for endomyocardial biopsy. Although the theoretical possibility exists that some of our patients suffered from coronary artery disease, the invasive exclusion of coronary stenosis in patients with a low pretest probability makes this rather unlikely.

The small sample size of our study requires further evaluation of the hypothesis with larger studies and the fact that this is not a prospective study weakens the methodological quality of our work.

The possible effect of medication on our findings cannot be fully ascertained since the control group was not subjected to the same medical treatment.

## Conclusion

A transient elevation of global myocardial T2 signal intensity is associated with a transient increase in left ventricular mass. Myocardial edema in the acute phase resolving at follow up could provide a potential explanation of our findings although other factors may contribute to this relation.

## Authors' contributions

AZ designed and coordinated the study, carried out most of the CMR-exams and drafted the manuscript. RW participated in the design of the study and provided large parts of the references cited in the manuscript. HAA performed the statistical analysis and proof-read the manuscript repeatedly. AR performed the image analysis. RD conceived of the study and participated in its coordination. JSM conceived of the study, participated in its design and helped with the revision of the manuscript. All authors read and approved the final manuscript.
